# Children’s dental panoramic radiographs dataset for caries segmentation and dental disease detection

**DOI:** 10.1038/s41597-023-02237-5

**Published:** 2023-06-14

**Authors:** Yifan Zhang, Fan Ye, Lingxiao Chen, Feng Xu, Xiaodiao Chen, Hongkun Wu, Mingguo Cao, Yunxiang Li, Yaqi Wang, Xingru Huang

**Affiliations:** 1 State Key Laboratory of Oral Diseases, National Clinical Research Center for Oral Diseases, West China Hospital of Stomatology, Sichuan University, Chengdu, 310000 China; 2 Division of Advanced Prosthetic Dentistry, Tohoku University Graduate School of Dentistry, Sendai, 310000 Japan; 3 Lishui University, School of Medicine, Hangzhou Geriatric Stomatology Hospital, Hangzhou Dental Hospital Group, Hangzhou, 310000 China; 4 School of Medicine and Health Sciences, Lishui University, Lishui, Zhejiang, 323000 China; 5 Hangzhou Dianzi University, Hangzhou, 310018 China; 6 College of Media Engineering, Communication University of Zhejiang, Hangzhou, 310018 China; 7 Department of Radiation Oncology, University of Texas Southwestern Medical Center, Dallas, Texas 75390 USA; 8 School of Electronic Engineering and Computer Science, Queen Mary University of London, Mile End Road, London, E1 4NS UK

**Keywords:** Image processing, Machine learning, Gum disease, Paediatric research

## Abstract

When dentists see pediatric patients with more complex tooth development than adults during tooth replacement, they need to manually determine the patient’s disease with the help of preoperative dental panoramic radiographs. To the best of our knowledge, there is no international public dataset for children’s teeth and only a few datasets for adults’ teeth, which limits the development of deep learning algorithms for segmenting teeth and automatically analyzing diseases. Therefore, we collected dental panoramic radiographs and cases from 106 pediatric patients aged 2 to 13 years old, and with the help of the efficient and intelligent interactive segmentation annotation software EISeg (Efficient Interactive Segmentation) and the image annotation software LabelMe. We propose the world’s first dataset of children’s dental panoramic radiographs for caries segmentation and dental disease detection by segmenting and detecting annotations. In addition, another 93 dental panoramic radiographs of pediatric patients, together with our three internationally published adult dental datasets with a total of 2,692 images, were collected and made into a segmentation dataset suitable for deep learning.

## Background & Summary

Dental disease not only causes serious oral problems but can also have adverse effects on other organs^1,2^. According to numerous studies, patients with periodontitis, dental caries, and other dental diseases have a significantly increased risk of coronary heart disease, myocardial infarction, cerebrovascular disease (such as stroke), and a higher risk of ischemic, hemorrhagic stroke, and cerebral ischemia^3^. The Global Burden of Disease Study 2019 estimates that nearly 3.5 billion people worldwide suffer from oral diseases, with dental caries in permanent teeth being the most^4^. Dental caries is one of the most common chronic diseases among people worldwide, and individuals are at risk of developing this dental disease throughout their lives. The disease develops in the crowns and roots of the teeth, and it may appear as aggressive tooth decay in early childhood^5^. Early childhood caries may lead to a more severe caries experience in adulthood, while a caries-free primary dentition is more likely to remain caries-free in the permanent dentition^6^. The disintegration of primary crowns due to severe caries may also lead to malnutrition and low body mass index in young children^7^. Globally, it is estimated that 200 million people suffer from permanent tooth decay and 520 million children suffer from milk tooth decay^4^ (https://vizhub.healthdata.org/gbd-results/healthdata). According to the research, the caries situation in children is increasing year by year. Therefore, the correct prevention and treatment of dental caries in young children have important medical value.

**Fig. 1:**
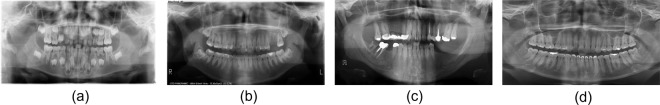
Different panoramic radiographs of teeth in children and adults: (**a**) from a 6-year-old pediatric patient; (**b**–**d**) are panoramic radiographs from three adult dental datasets, respectively.

The current treatment of children’s teeth relies heavily on the physician’s on-site judgment, where the physician will combine panoramic radiographs after the consultation to diagnose the patient’s dental disease and thus prescribe the right treatment. Among the 100 patients aged 2 to 13 years finally obtained from our dataset, 71.29% of them showed multiple teeth disease and cross disease, which poses a more serious challenge for doctors to comprehensively pinpoint the lesion and precisely plan the treatment. Designing a deep learning algorithm that effectively segments teeth and automatically analyzes diseases will greatly simplify the consultation process for doctors, reduce the rate of misclassification and missing disease detection, and thus improve the efficiency of medical work. Utilizing the existing effective deep learning network models can offer a practical framework for implementing the algorithm, but the training and testing of the model are inseparable from the data set. At present, there is no public data set for children’s teeth in the world, and the only three existing public data sets for adults’ teeth are not only different from children’s teeth in terms of the physiological structure, but also not suitable for caries segmentation and disease detection. Therefore, it is not scientific to use the existing adult dental dataset to conduct dental disease studies in children. There may be limitations in building a children’s dental dataset as follows^8^: (1) it is difficult to obtain patient data from medical institutions (2) it is often expensive to hire experts to annotate images (3) the importance of this field is not sufficiently appreciated. Some examples are shown in Fig. 1.

**Fig. 2:**
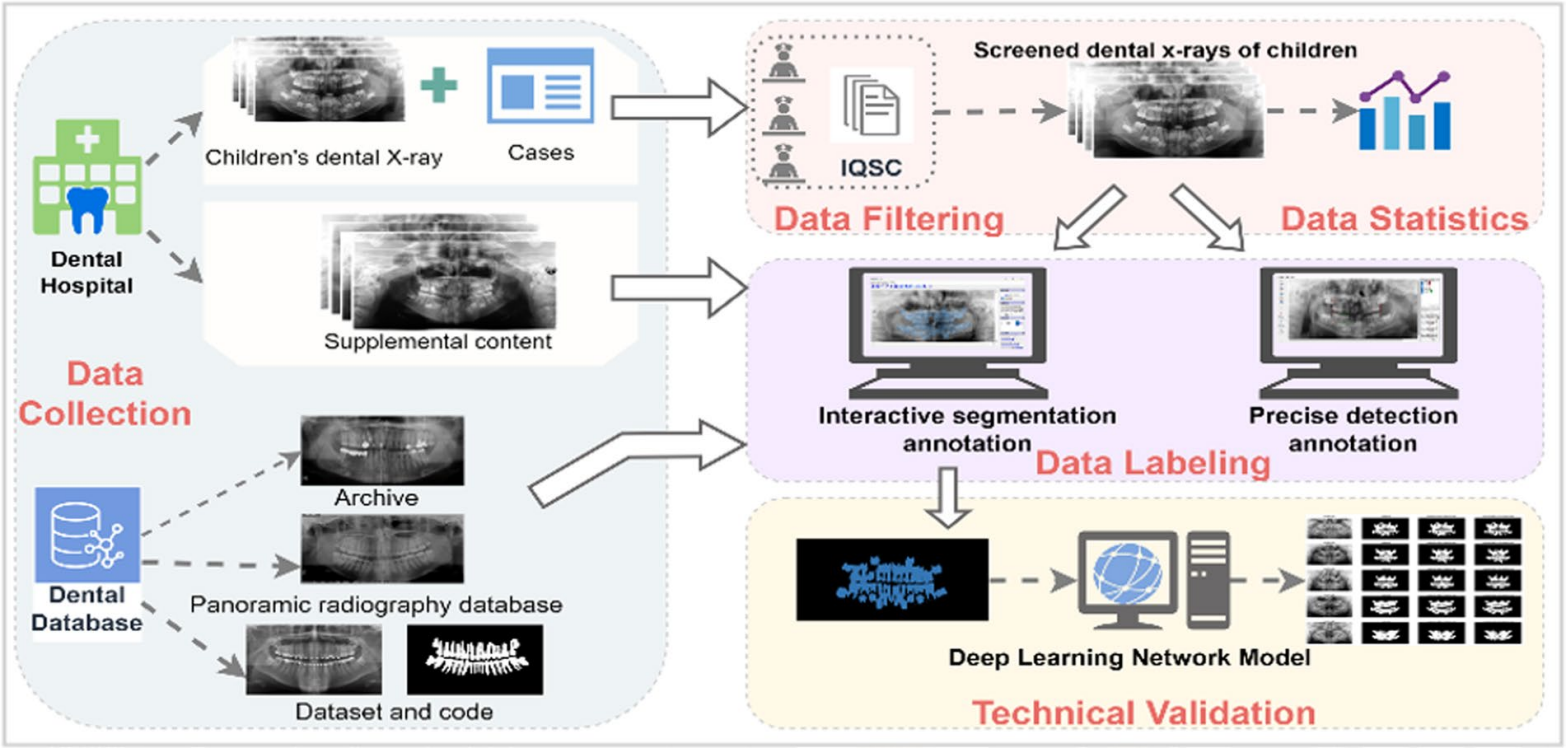
This figure is the core overview diagram of the article, which introduces the dataset production process, including data collection, data filtering and statistics, annotation work, and validation of the dataset using deep learning algorithms. The figure shows an overview of the core content of this article, including the dataset production process and validation.

Breaking through the limitations above, we obtained data from 106 patients aged 2 to 13 years old and 93 children’s dental panoramic radiographs and invited 7 dentists to participate in the production of the children’s dental dataset. We cleaned and screened the personal information of the obtained 106 dental panoramic radiographs, and finally obtained 100 high-quality images. With the help of image annotation software EISeg and LabelMe, we annotated the overall tooth structure segmentation and the accurate class-by-class detection of disease teeth, respectively, obtained the corresponding mask and divided the training set and test set by 7:3 to obtain the caries segmentation dataset and dental disease detection dataset. In addition, we annotated the collected 93 children’s dental panoramic radiographs with three cases of the adult dataset using caries segmentation to obtain a total of three cases of 2693 images adult dental dataset and one case of 93 children’s dental dataset respectively, which are publicly published in figshare as the supplementary material of children’s dental set^9^, and the core idea and process of the whole work will be shown by Fig. 2.

## Methods

**Fig. 3:**
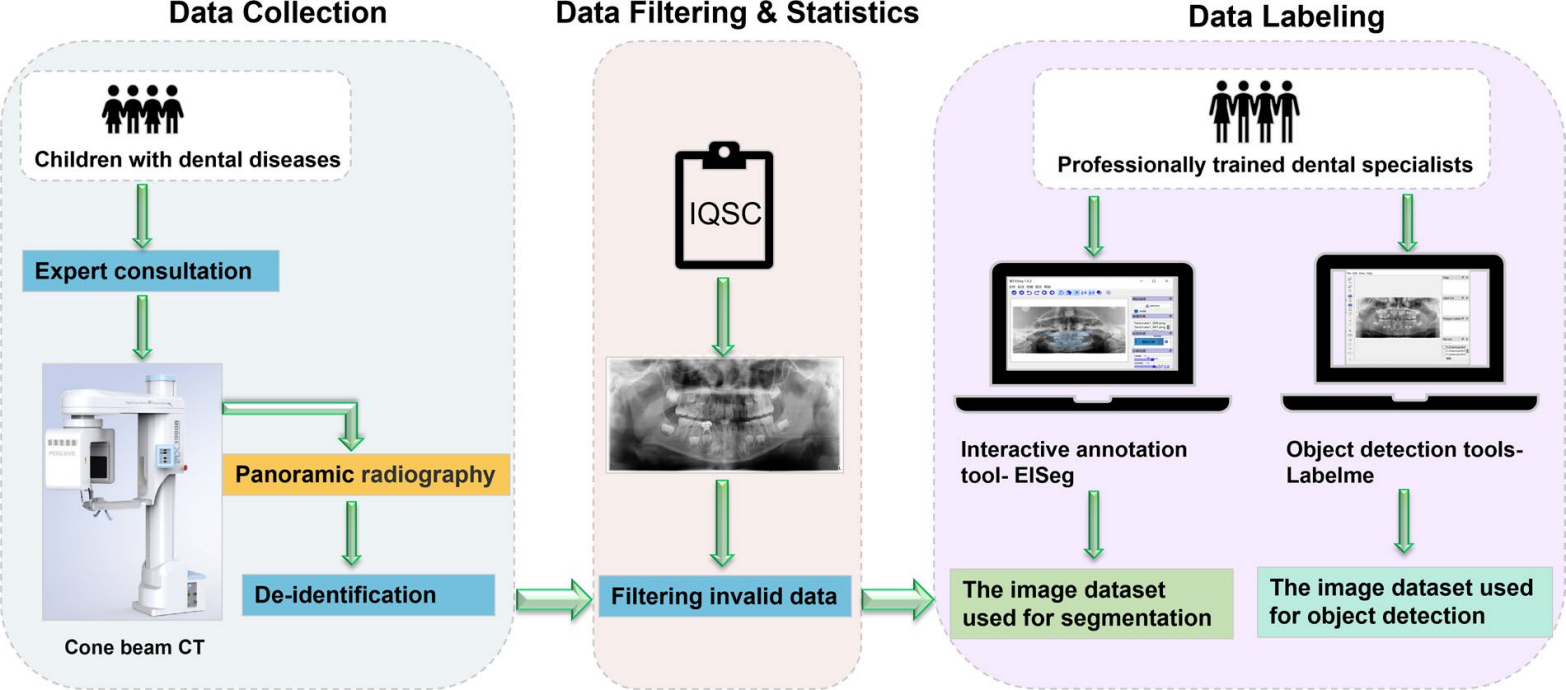
Production process of children’s dataset: The data came from 106 pediatric patients in Hangzhou Xiasha District Dental Hospital, and after expert consultation, patient data such as curved tomography were obtained by CBCT scanning. We eliminate all personal privacy information in the data, use the Pediatric CT Image Quality Scoring Standard (IQSC)^17^ to manually screen and exclude unqualified images, and annotate the resulting images in EISeg and Labelme respectively to obtain the corresponding data set.

**Fig. 4:**
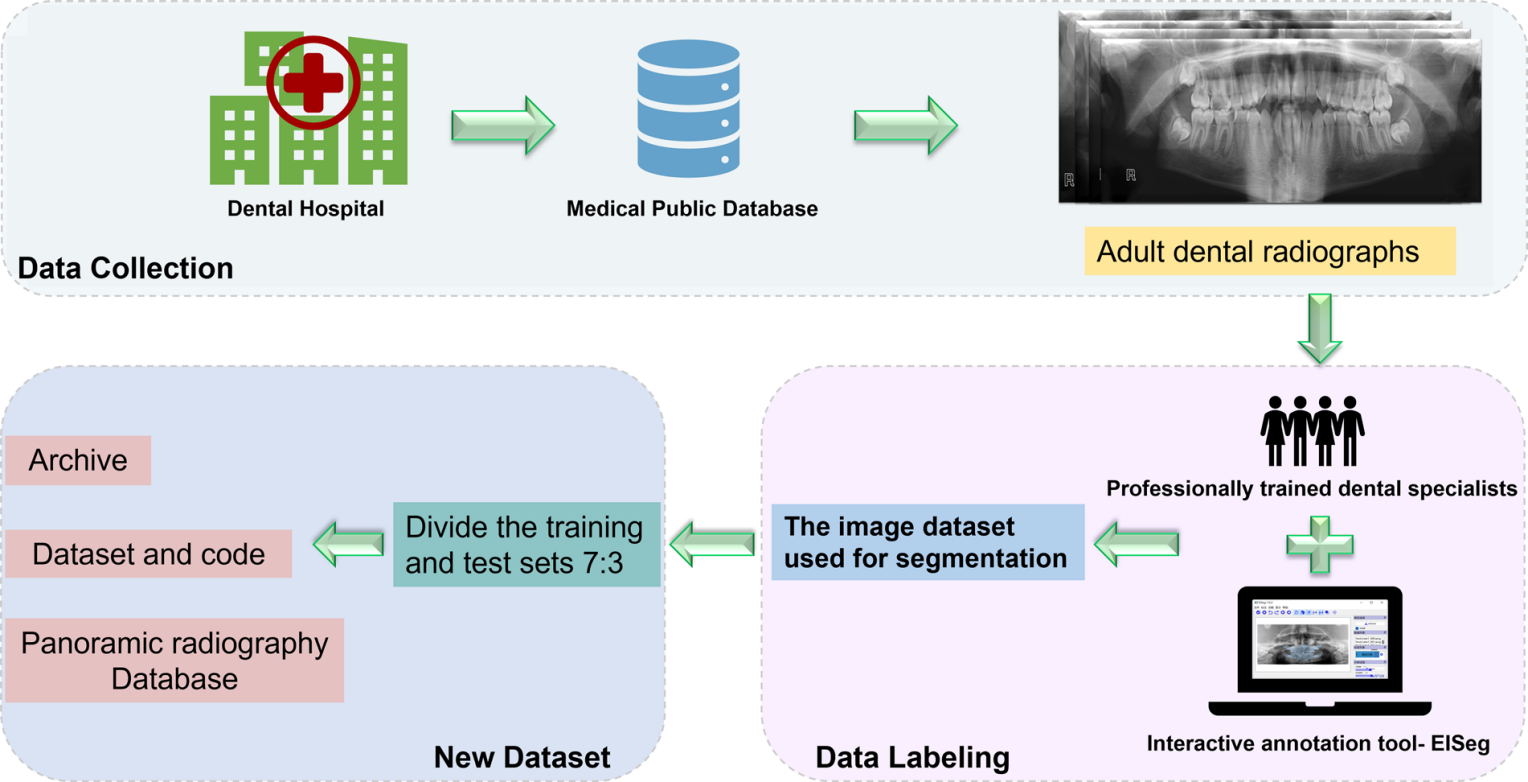
Production process of adult dental dataset: The basic characteristics of each dataset are given at the collection stage, including name, number of pictures, resolution, and the original purpose of the dataset. Experts will annotate the obtained data set for segmentation and annotation of the overall tooth structure and divide the data set.

We divided the production of the children’s dental dataset into three main stages, firstly, data collection, secondly, data filtering, and finally, data annotation, the main workflow of which can be seen in Fig. 3. In addition, we processed the collected adult dental dataset with similar data annotation and repartitioned the dataset to obtain three adult dental datasets with segmentation annotations, which are used to describe the production process in Fig. 4. The “Data Collection” section shows the basic characteristics of the datasets in tabular form.

### Data collection

**Fig. 5:**
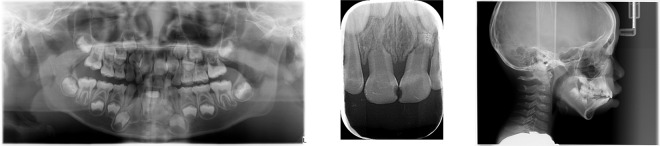
The figure shows three types of dental images we collected from hospitals, all of which are important tools for dentists to diagnose dental diseases. From left to right: dental panoramic radiographs, intraoral scan, CBCT.

The pediatric dental data set was obtained from cases of patients who visited the Hangzhou Xiasha Dental Hospital from March to June 2022, as well as panoramic radiographs from instrument scans at the time of the visit and intraoral scans and CBCT (see Fig. 5), for a total of 123 films. The study showed panoramic radiographs can be a valuable diagnostic tool for dental diseases, as they can capture the complete upper and lower jaw region on a single film. This method can also identify dental abnormalities, such as carious and periapical lesions, which may not be detectable during routine clinical examinations. It is most effective in detecting obstructive teeth and other anomalies due to its wider coverage^10^. Currently, dental studies using deep learning methods are based on panoramic radiographs^11–13^; therefore, our caries segmentation dataset as well as the dental disease detection dataset were produced for panoramic radiographs that corresponded one-to-one with the patient. In addition, intraoral scans showing the local details of the patient’s dental structure are presented as reference materials, and CBCT showing the full picture of the child’s dental structure in a three-dimensional form from the side will be published simultaneously as supplementary materials. In addition, we collected 93 panoramic radiographs of children’s teeth without cases as supplementary information, so there are 193 images in total in the segmented data set of children’s dental caries.

Another important task for us is to collect publicly available adult dental datasets and annotate them with segmentation. There are three international publicly available adult dental datasets, namely Dataset and code^14^, Panoramic radiography Database^15^, and Archive^16^, whose information features can be seen in Table 1. The images in the Dataset and code were acquired in part at the Diagnostic Imaging Center of the Southwest State University of Bahia (UESB) and in part by the Hangzhou Lishui Dental Hospital. 1978 images in total in Dataset and code^14^, including test set 1500 images and training set 478, where the content of the validation and training sets are kept consistent. This dataset has completed the work we expected to produce for the adult dental dataset, and the dataset itself has been provided with the original images and the annotated images generated by the segmentation, along with files describing the annotation information. The curved tomograms show the content of the treated adult dental panoramic images, where the orthodontic, filling and other medical treatments performed on the teeth can be seen. The panoramic radiography Database^15^ was proposed by Alireza Sadr *et al*. and was obtained from 598 randomly selected patients photographed for the realization of a study in the direction of image enhancement. The study direction was different and no obvious dental features were seen on the images, we collected only 598 dental panoramic radiographs for making the segmentation dataset. Archive^16^ consisted of anonymous and de-identified panoramic dental X-rays of 116 patients taken at Noor Medical Imaging Center in Qom, Iran, covering a wide range of dental diseases from healthy to partially and completely edentulous cases, two dentists manually segmented the mandibles of all cases. As we only required segmentation annotations for teeth and not mandibles, we only collected 116 panoramic radiographs to construct the segmentation dataset. In total, we gathered 2692 panoramic radiographs of adult dentition, out of which 714 required processing. However, there is an imbalance between the number of adult and children’s dental cases in our dataset. The smaller number of children’s cases is due to their relative scarcity. However, we are actively working on collecting new panoramic radiographs of children’s teeth to expand the dataset and mitigate this limitation. We have added a folder called “Children’s teeth - supplement” to our dataset, which houses all newly acquired and annotated panoramic radiographs of children’s teeth. We will continuously update this folder with new data as we collect and annotate additional radiographs.

**Table 1:** The basic characteristics of the three adult dental datasets collected from public databases.

Dataset	Number	Resolution	Caries segmentation	Feature
**Dataset and code**^14^	1978	1991 × 1127	Available	Dataset of adult teeth with segmentation annotations
**Panoramic radiography Database**^15^	598	2041 × 1024	N/A	Post-consultation adult panoramic radiographs
**Archive**^16^	116	2000 × 942	N/A	Adult dental dataset with mandibular segmentation labels
**Total**	**2692**			

### Data privacy

All patients included in the children’s dataset have given their consent for the use of their data for scientific research. The hospital has ensured that the confidentiality of their personal information will be maintained, and the consent of the children’s parents or guardians has been obtained and signed on documented. Upon receiving the data from the hospital, the doctors on our team took steps to anonymize the data, such as replacing patient names with numerical identifiers and removing any identifying features from the dental images. The images were then labeled with numerical codes, ensuring that the entire dataset does not contain any private information about the patient’s identity. To ensure that all identifiable patient information was removed, we established a team of five members to meticulously parse and manually review the data. Our dataset was scrutinized and approved by the ethics committee, as detailed in the collateral materials we provided.

### Data filtering and statistics

**Fig. 6:**
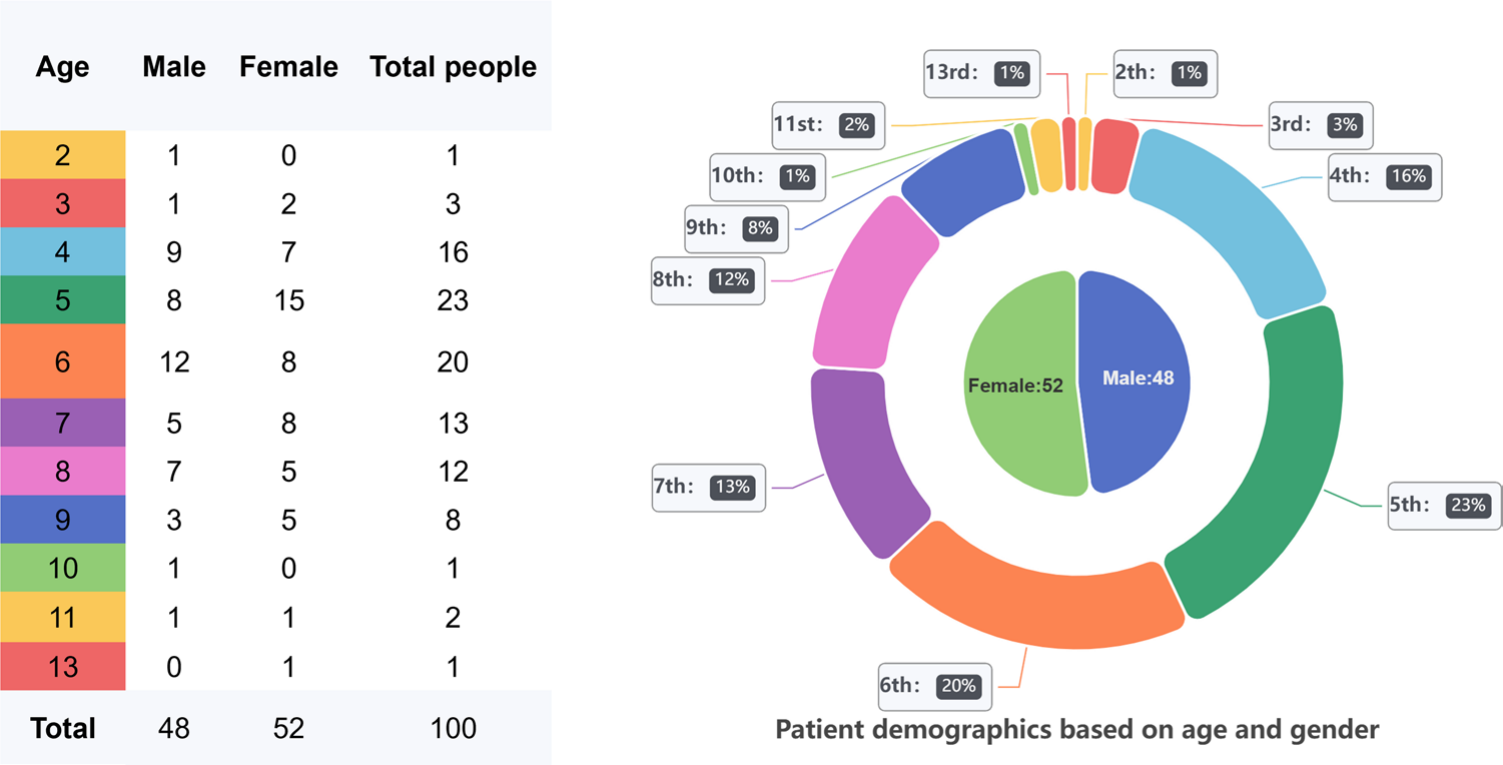
The table on the left presents the distribution of patients in the dataset by age group and sex in tabular form. The double-layer pie chart on the right shows the data characteristics in detail. The outer pie chart represents the distribution of people in each age group, and the label format is: age: proportion. The inner pie chart shows the ratio of men to women, of which 48 are boys and 52 are girls.

The adult dental dataset was screened operationally in the original paper and its details can also be referred to in the original paper^15,16^. Therefore, we screened 106 children’s dental data provided by hospitals to count the valid data. The main purpose of the screening was to filter out low-quality and misinformed children’s dental panoramic radiographs. To ensure the reasonable validity of the screening, we introduced the Image Quality Scoring Criteria for Pediatric CT (IQSC)^17^ as the main reference for screening.IQSC uses a scale of 0 to 4 to subjectively assess image quality, with 0 indicating the desired tooth structure or no features seen, 3 indicating whether the image quality is adequate, and 4 indicating that the image quality is higher than desired^17^. After screening, 5.67% of the images scored below 2. Therefore, we selected 100 panoramic radiographs with image quality in the range of 2 to 4 for the task. After selecting 100 panoramic radiographs, we combined the patient’s basic personal information provided on the case and counted the data according to age and gender, and the results of the statistics are visually displayed in Fig. 6.

**Fig. 7:**
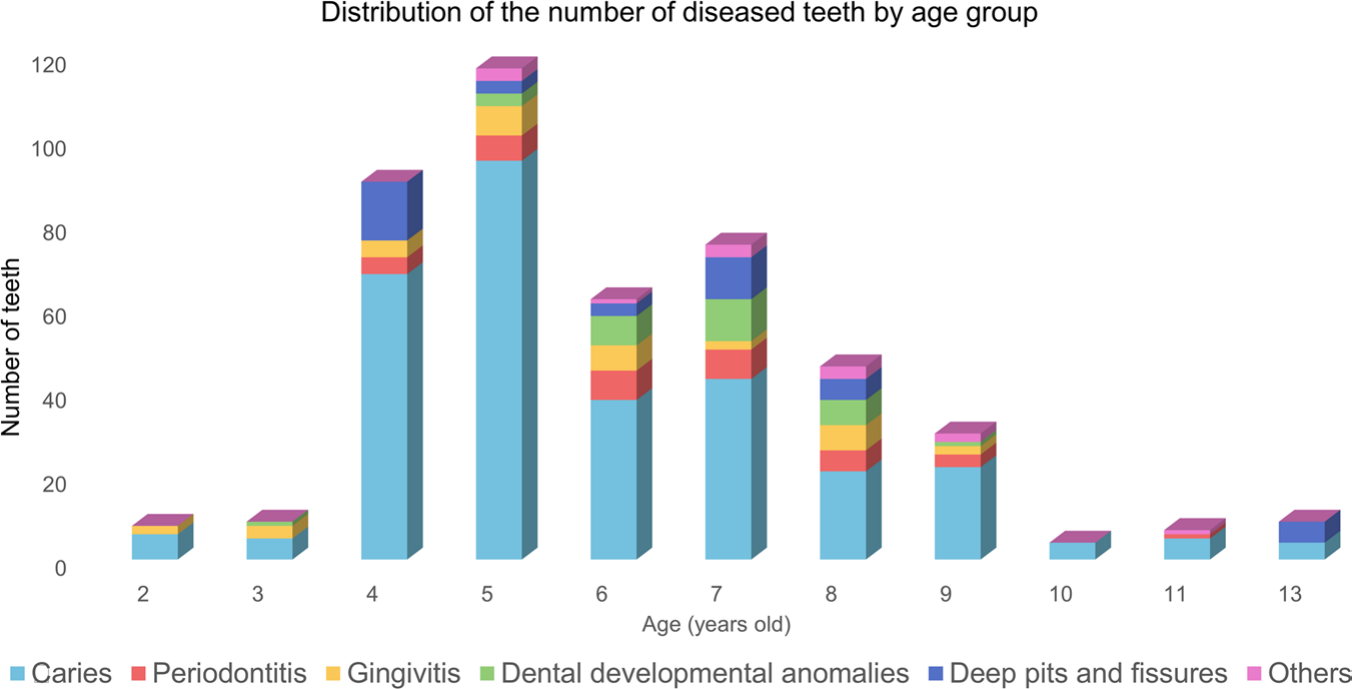
This figure visualizes the data in Table 2 in the form of a clustered bar chart combined with a pie chart. The horizontal axis of the bar chart represents the age of the patient, and the vertical axis indicates the number of teeth suffering from the disease, which is categorized and counted according to the legend. The pie chart at the top right shows the percentage of patients suffering from each type of dental disease.

Dental caries, pulpal and periapical lesions, dental trauma, developmental abnormalities, and oral habits are the most common dental diseases among children^7^, where dental developmental abnormalities usually include morphological, structural, numerical, and eruption abnormalities^18^, as reflected in the case-by-case review. We found that the most common dental diseases in the children, in this case, included dental caries, periapical periodontitis, pulpitis, deep sockets, and dental developmental abnormalities, in addition to other conditions such as pigmented teeth and dental examinations, from which the labels for dental disease detection were derived. To better count the probability of occurrence of various dental diseases, we counted the number of teeth with each type of disease present in each age group, and the results of the statistics can be seen in Table 2 and Fig. 7.

**Table 2:** Statistics on the number of teeth for each type of disease that occur in each age group.

Age	Caries	Periodontitis	Gingivitis	Dental developmental anomalies	Deep pits and fissures	Others	Total number of diseased teeth
2	6(75.00%)	0	2(25.00%)	0	0	0	8
3	5(55.55%)	0	3(33.33%)	1(11.12%)	0	0	9
4	68(75.56%)	4(4.44%)	4(4.44%)	0	14(15.56%)	0	90
5	95(81.20%)	6(5.14%)	7(5.98%)	3(2.56%)	3(2.56%)	3(2.56%)	117
6	38(61.29%)	7(11.29%)	6(9.68%)	7(11.29%)	3(4.84%)	1(1.61%)	62
7	43(57.33%)	7(9.33%)	2(2.68%)	10(13.33%)	10(13.33%)	3(4.00%)	75
8	21(45.67%)	5(10.87%)	6(13.04%)	6(13.04%)	5(10.87%)	3(6.52%)	46
9	22(73.33%)	3(10.00%)	2(6.67%)	1(3.33%)	0	2(6.67%)	30
10	4(100%)	0	0	0	0	0	4
11	5(71.42%)	1(14.29%)	0	0	0	1(14.29%)	7
13	4(44.44%)	0	0	0	5(55.56%)	0	9
Total	311(68.05%)	33(7.22%)	32(7.00%)	28(6.13%)	40(8.76%)	13(2.84%)	457

### Data annotation

The data annotation work was divided into two categories: (a) overall segmentation of tooth structure and (b) detection annotation by dental disease classification. We performed the two types of operations as above on the children’s dental dataset, i.e., segmentation annotation and detection annotation of images in parallel, and overall segmentation of tooth structure on 714 adult dental images. Since the processing of segmentation annotation is not significantly different on the adult and child datasets, we describe the annotation of child dental tomograms as an example.

### EISeg

The overall segmentation annotation of dental structures is mainly performed in EISeg (Efficient Interactive Segmentation). EISeg gets rid of the disadvantages of other software that relies heavily on manual annotation, and its core is an interactive segmentation algorithm implemented based on Baidu’s deep learning framework Flying Paddle, which greatly improves the segmentation efficiency and accuracy with the help of an interactive segmentation model accuracy^1^. When applying its specific model for the medical imaging domain, the tool can quickly generate highly accurate segmentation masks, and users can manually adjust the region boundaries to optimize the segmentation accuracy. This feature is especially beneficial for medical professionals who require precise and rapid segmentation of images.

**Fig. 8:**
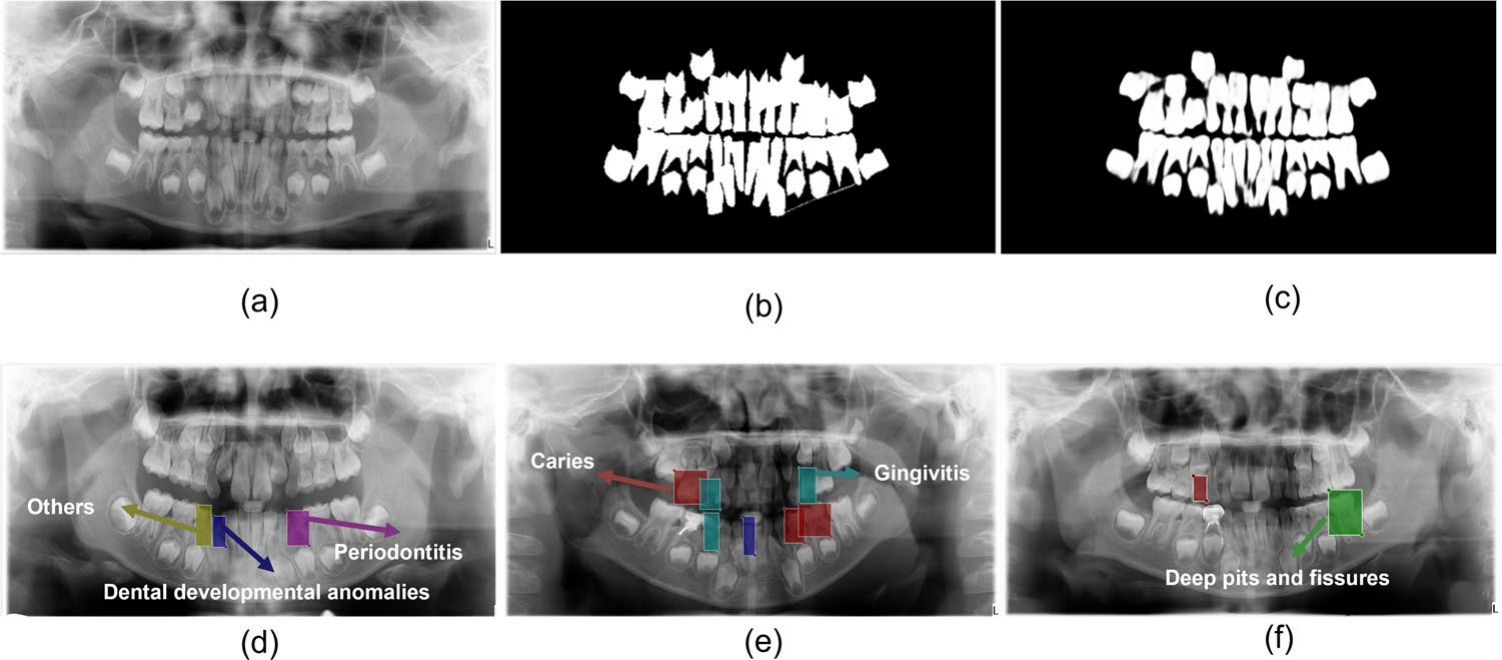
(**a**) indicates the original image, (**b**) is obtained by segmentation annotation software, and (**c**) is the prediction mask obtained by a deep learning network. (**d**–**f**) simulate the process of dental disease detection annotation, with colored rectangular boxes indicating which dental disease the tooth is suffering from.

We trained three dentists to annotate the segmentation of the overall tooth structure on EISeg and checked the results of their trial annotation, and finally, all three dentists could annotate correctly. Since EISeg has a limit on the size of the input images, we modified all images to a uniform resolution of 2000*942 before annotation, and all images were anonymized. The three dentists were annotated with the number of images 33, 33, and 34, and the single label category of the data was set to “teeth”, and the model used for annotation was lightweight, and the original image and the annotated mask can be seen in Fig. 8. If there is no objection, the segmentation and annotation of the overall tooth structure will be completed.

### LabelMe

The annotation of the detection by dental disease classification is labeled for children’s dental susceptibility, with label categories including (1) caries (2) periapical infection (3) pulpitis (4) deep sulcus (5) dental developmental abnormalities (6) others. The annotation of the detection dataset is mainly applicable to the target detection task in the field of computer vision, where the annotator needs to pinpoint the lesion as well as diagnose the disease type, and this annotation work is significantly more demanding and complex. To improve the efficiency of the annotation work, this annotation work is mainly performed in LabelMe^2^, an online web-based annotation tool that allows easy image annotation and instant sharing of these annotations. A total of six dental experts were involved in the design and implementation of the annotation work, and they systematically learned the use of LabelMe to understand the meaning of the six types of labels. Four of the experts, each randomly assigned 25 anonymous images, completed the first round of the image annotation task independently. The other two experts reviewed the labeled images and the accuracy of the labeling results. When the review found ambiguities in the results, the six reviewers discussed and discussed, and the resulting unified opinion was used as the final result. The images tested are displayed in Fig. 8, and the resulting annotation information is saved in the form of JSON files.

After getting the annotation information of the dataset, we divide the two types of dataset images into training and test sets in the ratio of 7:3, both containing the original images and the corresponding annotation information (segmentation annotations are mask files, and testing annotations are JSON files).

## Data Records

**Fig. 9:**
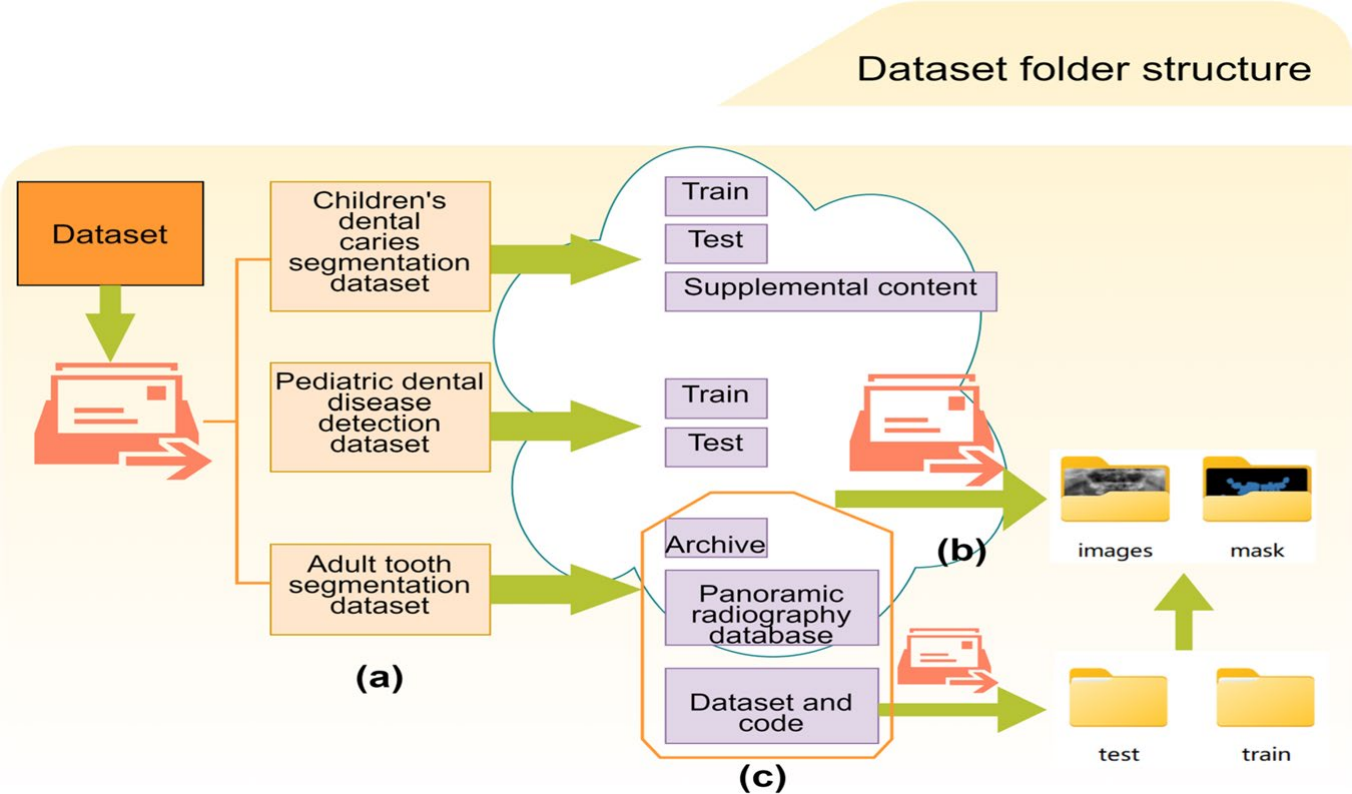
Dataset folder structure: (**a**) shows the overall structure, (**b**) shows the file content and structure of the “Children’s Dental Caries Segmentation Dataset”, and (**c**) shows the file organization of the “Adult Dental Segmentation Dataset”.

The Children’s dental panoramic radiographs dataset is stored at figshare^9^. We provide all datasets as mentioned above, including the caries segmentation dataset as well as the dental disease detection dataset, and the list of provided datasets is shown in Table 3. The pediatric dental datasets have been compressed and submitted to the (stored website) for viewing and downloading by a wide range of scholars and researchers. The uncompressed file includes three datasets, which are the “Child Dental Caries Segmentation Dataset”, “Child Dental Disease Detection Dataset” and “Adult Dental Segmentation Dataset” (see Figure (a) of Fig. 9). The “Child Dental Caries Segmentation Dataset” and “Child Dental Disease Detection Dataset” contain “Train” and “Test “, which are used to record the training set and test set division of the dataset, the training set or test set includes the original image and annotation information (segmentation annotation is mask file, detection annotation is JSON file), and its structure can be seen in part (b) of Fig. 9. The training set of 70 images was divided into 5 groups of 14 images each, and the test set of 30 images was divided into 2 groups of 15 images each, named in the format of “cateX_0XX”. The “ Adult tooth segmentation dataset” collects the file information of three cases of adult teeth after segmentation and annotation, and its internal structure is similar to that of the “Child tooth caries segmentation dataset”, and its structure is shown in part (c) of Fig. 9.

**Table 3:** Summary and list of provided dataset features.

	Dataset	Number	Resolution	Caries segmentation	Dental disease detection	Total
**Child**	Children Tooth Dataset	100	2000 × 942	Available	Available	2885
	Children’s Dental Supplement Dataset	93	2000 × 942	Available	N/A	
**Total**				193		
**Adult**	Dataset and code^14^	1978	1991 × 1127	Available	N/A	
	Panoramic radiography Database^15^	598	2041 × 1024	Available	N/A	
	Archive^16^	116	2000 × 942	Available	N/A	
**Total**				2692

## Technical Validation

### Application prospects and fields

The emergence of deep convolutional networks in 2012 and U-Net in 2015 for medical image segmentation has led to a surge in the adoption of machine learning and deep learning algorithms in medical image analysis. Consequently, applying deep learning algorithms to precisely segment teeth and automate the detection and analysis of dental diseases has become a prominent research area in dentistry. Gil Jader^19^ *et al*. proposed a Mask R-CNN (mask R-CNN) based segmentation system to segment the whole structure of adult teeth, Lee^12^ *et al*. used GoogleLeNet Inception v3 CNN network and transfer learning to detect and diagnose dental caries, and Chen^20^ *et al*. proposed a TRANSFORMER as an encoder to TransUNet proposed by Chen^20^ can also be used for medical image segmentation, and the U-Net-like group transformer network (GT U-Net) proposed by Li *et al*.^21^ further solves the blurred boundary problem of tooth root segmentation. These applications are based on adult dental datasets, and to our knowledge, there are no applications of deep learning algorithms based on children’s dental datasets.

### Segmentation experimental verification

In order to demonstrate the practical utility of the children’s dental segmentation dataset presented in this study, and to provide researchers with potential applications, we conducted experiments using well-established deep learning networks commonly utilized in medical image segmentation, namely U-Net, R2 U-Net, PSPNet, and DeepLab V3. Experiments using the networks as proposed above can visually and precisely show the accurate validity of the dataset by the performance evaluation metrics commonly used in the image segmentation field, and we used five evaluation metrics, including Recall, Specificity, Accuracy (ACC), Intersection over Union (IoU), Dice coefficient (Dice), and the mean and standard deviation on different datasets were calculated.

- **U-Net**^22^. The U-Net successfully breaks the drawback of previous deep networks that require thousands of annotated training samples and proposes a training strategy that relies on data augmentation to use annotated samples more effectively^22^.
- **R2 U-Net**^23^. A residual convolutional neural network (RRCNN) based on the U-Net model, known as R2U-Net, with an attention gate, was proposed by Alom *et al*. This architecture builds upon the strengths of U-Net, residual network, and RCNN, while also incorporating an attention gate mechanism. This modification enhances the model’s ability to focus on relevant image features and suppress irrelevant ones, resulting in improved performance for medical image segmentation tasks.
- **PSPNet**^24^. Pyramid Scene Parsing Network (PSPNet) is applied to scene parsing tasks and is often used to do semantic segmentation, which introduces more contextual information in the semantic segmentation algorithm achieved by global mean pooling manipulation and feature fusion to avoid many missegmentations.
- **DeepLab V3+**^25^. DeepLab v3+ adds a simple and effective decoder module to DeepLab v3 to correct the segmentation results, especially the target boundaries, and further explores the combination of Xception and depth-separable convolution with ASPP and decoder modules to produce a faster and stronger network.

We conducted the above four experiments on the children’s dental data set, the adult dental data set, and the combined data set, respectively, with a uniform batch size of 4, an image size of 512 × 512, and a loss function set to cross-entropy. The reason why we organized the experiment of adding the adult dataset to the children’s dataset was to demonstrate that the adult dataset, although greatly expanding the number of children’s datasets, did not improve the experimental results of the children’s dental dataset in terms of experimental effects. Therefore, it is not reasonable to directly apply the algorithm obtained from the study of adult dental dataset to the study of children’s teeth because of the difference in the physiological structure of adult and children’s teeth. 163 training sets and 30 test sets were used for the children’s dental data set (93 supplemental data sets were added to the training set). For the adult dental dataset, 1500 training sets are taken from the training set of Data and code, and 276 test sets are taken from the test set of Data and code; the combination of the two is to merge the training set and the test set respectively.

### Validation results

**Fig. 10:**
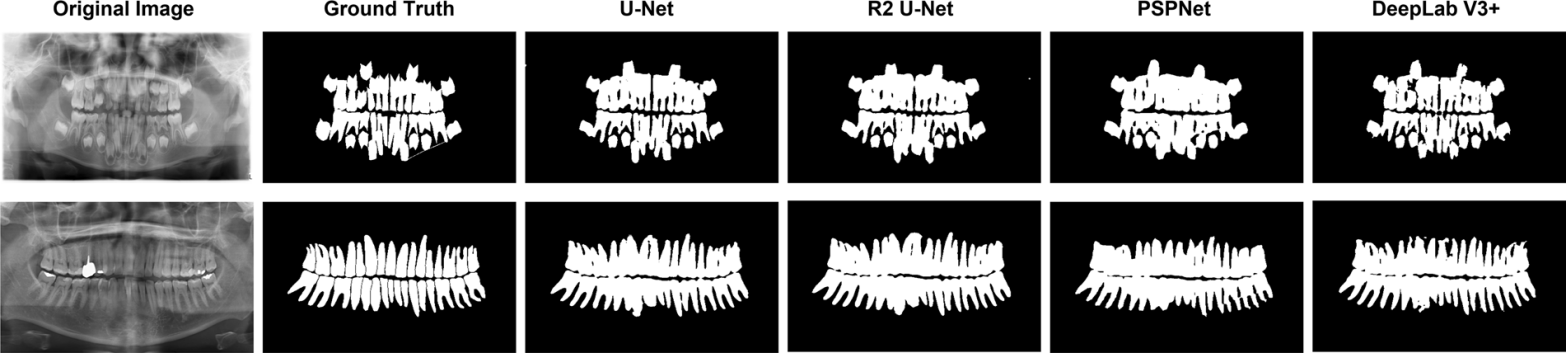
Shown are two different sets of images, from left to right: the original image, Ground-truth, and the predicted mask obtained from the three networks.

We present the experimental results evaluation metrics in Tables 4–6, respectively, and Fig. 10 shows the prediction masks obtained from the experiments with the child dataset and the adult dataset, respectively, which visually shows that the segmented dataset can get still better results after training into the network. Taking the experimental evaluation metrics obtained based on the children’s dental dataset as an example, our dataset obtained IoU mean value of 0.8389, Recall mean value of 0.92, and accuracy mean value of 00.9710 on U-Net and performed well in other networks and other metrics. The experimental data, as above, all show that our proposed dataset can be well applied to deep learning algorithms.

**Table 4:** Experimental results for the child datasets, trained exclusively on the children’s dental dataset (163 training images, 30 test images).

	Recall		Specificity		ACC		IOU		Dice	
	mean	std	mean	std	mean	std	mean	std	mean	std
U-Net^22^	0.9200	0.0375	0.9803	0.0057	0.9710	0.0052	0.8387	0.0300	0.9120	0.0182
R2 U-Net^23^	0.8854	0.0679	0.9840	0.0055	0.9675	0.0081	0.8247	0.0563	0.9027	0.0375
PSPNet^24^	0.8875	0.0198	0.9856	0.0049	0.9683	0.0065	0.8324	0.0221	0.9083	0.0132
Deeplab V3+^25^	0.9486	0.0162	0.9701	0.0069	0.9670	0.0058	0.8121	0.0259	0.8961	0.0158

Evaluation metrics include Recall, Specificity, Accuracy, Intersection over Union (IOU), and Dice index, with mean and standard deviation (std) values provided.

**Table 5:** Experimental results for the adult datasets, trained exclusively on the adult dental dataset (1500 training images, 276 test images).

	Recall		Specificity		ACC		IOU		Dice	
	mean	std	mean	std	mean	std	mean	std	mean	std
**U-Net**^22^	0.9459	0.0231	0.9795	0.0119	0.9719	0.0083	0.8858	0.0274	0.9392	0.0162
**R2 U-Net**^23^	0.9351	0.0268	0.9838	0.0112	0.9724	0.0079	0.8892	0.0264	0.9411	0.0156
**PSPNet**^24^	0.9164	0.0175	0.9827	0.0044	0.9670	0.0063	0.8693	0.0201	0.9299	0.0122
**Deeplab V3+**^25^	0.9465	0.0218	0.9721	0.0130	0.9665	0.0088	0.8639	0.0284	0.9267	0.0171

Evaluation metrics include Recall, Specificity, Accuracy, Intersection over Union (IOU), and Dice index, with mean and standard deviation (std) values provided.

**Table 6:** Experimental results for the combined adult and child datasets, trained on a merged dataset consisting of both children’s and adult dental datasets (1663 training images, 306 test images).

	Recall		Specificity		ACC		IOU		Dice	
	mean	std	mean	std	mean	std	mean	std	mean	std
**U-Net**^22^	0.9434	0.0261	0.9795	0.0114	0.9718	0.0080	0.8812	0.0310	0.9365	0.0183
**R2 U-Net**^23^	0.9300	0.0367	0.9839	0.0108	0.9719	0.0081	0.8828	0.0364	0.9373	0.0223
**PSPNet**^24^	0.9136	0.0197	0.9830	0.0046	0.9671	0.0063	0.8657	0.0230	0.9278	0.0138
**Deeplab V3+**^25^	0.9467	0.0213	0.9719	0.0125	0.9666	0.0086	0.8588	0.0321	0.9237	0.0192

Evaluation metrics include Recall, Specificity, Accuracy, Intersection over Union (IOU), and Dice index, with mean and standard deviation (std) values provided.

## Usage Notes

Readers who wish to download the dataset may do so at figshare^9^. We hope that this published dataset will be available to more researchers and encourage more authors to publish their optimized codes and models, which will contribute to the development and advancement of the field of pediatric dental disease.

## Data Availability

The code used for Technical Validation is public code and can be found in the original paper. The “Dataset and code^14^” dataset is available in our uploaded dataset folder, and the remaining two cases of the original adult dental dataset are available for download on Archive^16^ Panoramic radiography Database^15^. In addition, the annotation software referenced to produce the dataset is open source on EISeg^1^ and LabelMe^2^, respectively.
