# Publisher Correction: Children’s dental panoramic radiographs dataset for caries segmentation and dental disease detection

**DOI:** 10.1038/s41597-023-02795-8

**Published:** 2023-12-08

**Authors:** Yifan Zhang, Fan Ye, Lingxiao Chen, Feng Xu, Xiaodiao Chen, Hongkun Wu, Mingguo Cao, Yunxiang Li, Yaqi Wang, Xingru Huang

**Affiliations:** 1 State Key Laboratory of Oral Diseases, National Clinical Research Center for Oral Diseases, West China Hospital of Stomatology, Sichuan University, Chengdu, 310000 China; 2 Division of Advanced Prosthetic Dentistry, Tohoku University Graduate School of Dentistry, Sendai, 310000 Japan; 3 Lishui University, School of Medicine, Hangzhou Geriatric Stomatology Hospital, Hangzhou Dental Hospital Group, Hangzhou, 310000 China; 4 School of Medicine and Health Sciences, Lishui University, Lishui, Zhejiang, 323000 China; 5 Hangzhou Dianzi University, Hangzhou, 310018 China; 6 College of Media Engineering, Communication University of Zhejiang, Hangzhou, 310018 China; 7 Department of Radiation Oncology, University of Texas Southwestern Medical Center, Dallas, Texas 75390 USA; 8 School of Electronic Engineering and Computer Science, Queen Mary University of London, Mile End Road, London, E1 4NS UK

**Keywords:** Image processing, Machine learning, Gum disease, Paediatric research, Image processing, Machine learning, Gum disease, Paediatric research

Correction to: *Scientific Data* 10.1038/s41597-023-02237-5, published online 14 June 2023

In this article the affiliation details for Yifan Zhang were incorrectly given as ‘Hangzhou Dianzi University, Hangzhou, 310018, China’. The original article has been corrected.

